# SUMO1 Affects Synaptic Function, Spine Density and Memory

**DOI:** 10.1038/srep10730

**Published:** 2015-05-29

**Authors:** Shinsuke Matsuzaki, Linda Lee, Erin Knock, Tharan Srikumar, Mikako Sakurai, Lili-Naz Hazrati, Taiichi Katayama, Agnieszka Staniszewski, Brian Raught, Ottavio Arancio, Paul E. Fraser

**Affiliations:** 1 Tanz Centre for Research in Neurodegenerative Diseases, University of Toronto, 60 Leonard Avenue, Toronto, M5T 2S8, Ontario; 2 Molecular Brain Science, United Graduate School of Child Development, Osaka University, 2-2 Yamadaoka, Suita, Osaka, 565-0871, Japan; 3 Molecular Research Center for Children’s Mental Development, United Graduate School of Child Development, Osaka University, 2-2 Yamadaoka, Suita, Osaka, 565-0871, Japan; 4 Department of Anatomy and Neuroscience, Graduate School of Medicine, Osaka University, 2-2 Yamadaoka, Suita, Osaka, 565-0871, Japan; 5 Department of Pathology and Cell Biology and Taub Institute for Research on Alzheimer’s Disease and the Aging Brain, Columbia University, 630 W 168th St.New York, 10032, NY; 6 Ontario Cancer Institute, University Health Network; 7 Departments of Medical Biophysics, Laboratory Medicine and Pathobiology University of Toronto

## Abstract

Small ubiquitin-like modifier-1 (SUMO1) plays a number of roles in cellular events and recent evidence has given momentum for its contributions to neuronal development and function. Here, we have generated a SUMO1 transgenic mouse model with exclusive overexpression in neurons in an effort to identify *in vivo* conjugation targets and the functional consequences of their SUMOylation. A high-expressing line was examined which displayed elevated levels of mono-SUMO1 and increased high molecular weight conjugates in all brain regions. Immunoprecipitation of SUMOylated proteins from total brain extract and proteomic analysis revealed ~95 candidate proteins from a variety of functional classes, including a number of synaptic and cytoskeletal proteins. SUMO1 modification of synaptotagmin-1 was found to be elevated as compared to non-transgenic mice. This observation was associated with an age-dependent reduction in basal synaptic transmission and impaired presynaptic function as shown by altered paired pulse facilitation, as well as a decrease in spine density. The changes in neuronal function and morphology were also associated with a specific impairment in learning and memory while other behavioral features remained unchanged. These findings point to a significant contribution of SUMO1 modification on neuronal function which may have implications for mechanisms involved in mental retardation and neurodegeneration.

SUMO, a small ubiquitin-like modifier, is covalently attached by the formation of a reversible isopeptide bond between its C-terminal diglycine motif and the side chain of a lysine residue on a target protein. The conjugation is carried out via a series of enzymatic reactions by SUMO-specific enzymes, which can discriminate between SUMO isoforms^1^. Three primary SUMO paralogs (SUMO1, 2 and 3) have been identified in mammals. Their expression is to some extent cell-type specific and they display predominant, but not exclusive, subcellular localizations^2^,^3^. Mass spectrometry analyses have demonstrated the potential of these three isoforms to form poly-SUMO chains on internal lysines^1^.

SUMOylation is tightly regulated through spatial and temporal expression of SUMO proteins and the conjugation machinery. This regulation is crucial for signaling pathways involved in essential molecular and biological processes, from development to senescence^4^,^5^. This biological decline, or aging, is known to be a major risk factor for several neurodegenerative diseases. Cumulatively, these reports are consistent with the role of SUMOylation in normal aging as well as in the etiology of a number of neuropathological disorders^6^,^7^,^8^. SUMOylation has been implicated in a variety of neuronal pathways including synapse formation, synaptic transmission, excitability as well as axonal trafficking and axonal guidance^7^,^9^,^10^,^11^. A decrease in SUMO-modified proteins and a redistribution of SUMO enzymes to dendritic sites have been reported during the maturation of neurons^12^,^13^. Reports also suggest a neuroprotective role of SUMOylation in brain injuries caused by ischemia and also oxidative stress^14^,^15^.

In this study, we have generated and characterized a neuron-specific SUMO1 transgenic (Tg) mouse model. A proteomics screen was performed and a significant number of SUMO1 conjugate candidates were identified. Impairment of neuronal functions resulted in memory and learning defects. Altogether, this study paves the way for a better understanding of the role of SUMOylation in neuronal functions and dysfunctions.

## Results

### Characterization of SUMO1 Transgenics

**Figure 1:**
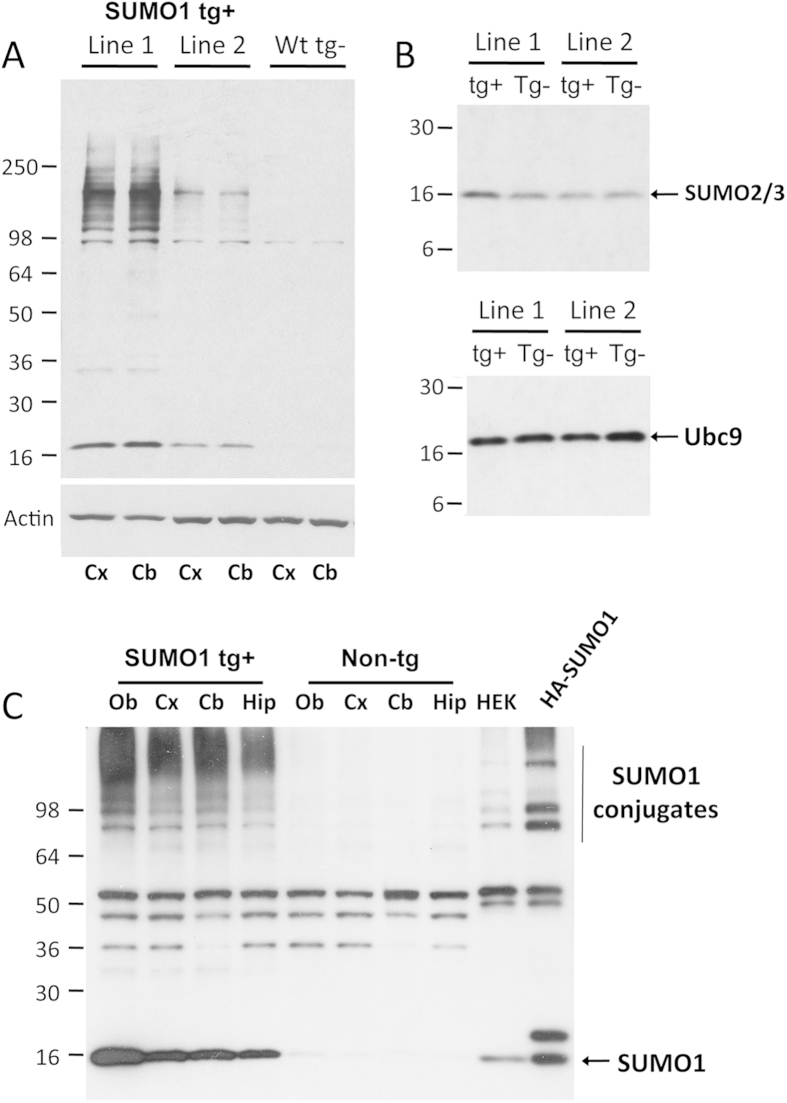
Development and characterization of SUMO1 mouse transgenic lines. (**A**) Immunoblotting of SUMO1 Tg founder lines shows relative levels of SUMO1 monomers and high molecular weight conjugates as compared to non-Tg mice (actin was used as a loading control). (**B**) Immunoblotting for SUMO2/3 and UBC9 in the SUMO1 transgenic lines indicated there were no significant changes in other SUMO-related proteins. (**C**) Regional expression of SUMO1 transgene as compares to non-Tg mice displays broad distribution in brain regions such as the olfactory bulb (Ob), cortex (Cx), cerebellum (Cb) and hippocampus (Hip). HA-tagged SUMO1 was used to confirm specificity.

In an effort to determine the effects of SUMOylation within neurons in an *in vivo* setting, an over-expressing full-length human SUMO1 Tg mouse model was generated using the prion cos-tet promoter. The prion promoter is a neuronal housekeeping gene and this vector directs position-independent integration that results in primarily expression in CNS neurons and to a more limited extent in astrocytes 16. Oocyte injection of purified transgene fragments produced two founder lines with whole brain expression levels that were elevated as compared to non-Tg animals (Fig. 1A). The lines differed in their relative SUMO1 expression with one model (Line 1) displaying higher increases in the unconjugated monomer and SUMO1-modified proteins than the second line (Line 2). This Tg model (Line 1) was investigated further to identify neuronal conjugation target proteins as well as the consequences on neuronal morphology and function. Immunoblotting was performed using a rabbit polyclonal that was generated against a peptide antigen specific for the C-terminus of human SUMO1 (Fig. S1) and verified through the use of commercially-available SUMO1 antibodies. The polyclonal antibody was developed in our laboratory to allow for the production of large quantities of affinity-purified material that could be used for proteomic studies (see below).

The effects of SUMO1 expression in the Tgs on other SUMO-related proteins were also investigated. Western blotting for SUMO2/3 from total brain lysates indicated there was no significant difference in the lower expression Tgs as compared to their non-Tg littermates (Fig. 1B). The high expressing line did show a slight increase in SUMO2/3 that may reflect decreased turnover and/or induced expression but the levels were not dramatically different when compared to non-Tg animals. Examination of Ubc9 by immunoblotting indicated similar static levels in Tgs and non-Tgs suggesting the total amount of Ubc9 was unchanged (Fig. 1B). Levels of the SUMO1 E1 activating enzyme (SAE1) were also examined by immunoblotting which displayed comparable levels in SUMO1 Tg and non-Tg mice (Fig. S2). Similarly, no differences were observed for the SUMO protease, SENP1, in these animals (data not shown). These findings suggest that the elevated SUMO1 expression had relatively little effect on the other SUMO proteins or the conjugation pathway elements. Tg and non-Tg brains were dissected and probing for SUMO1 demonstrated comparable levels in the cortex, cerebellum and hippocampus for both monomeric and conjugated SUMO1 (Fig. 1C).

**Figure 2:**
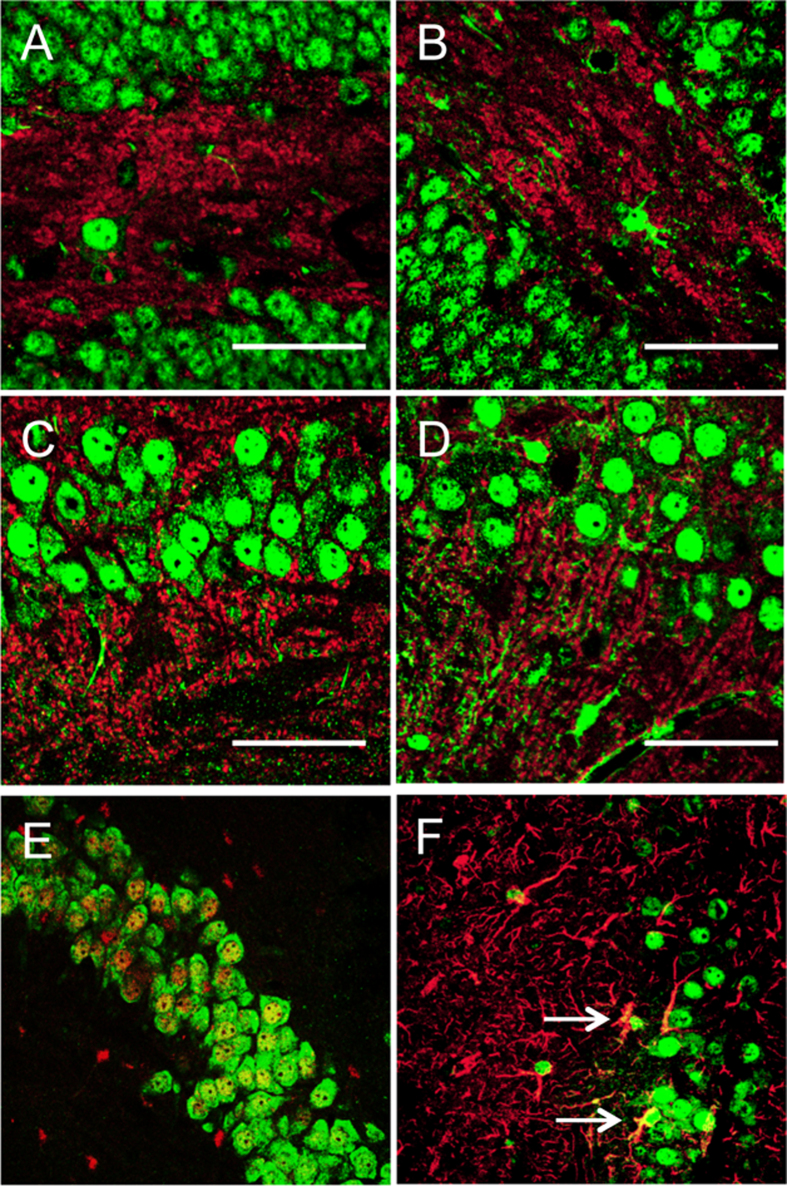
SUMO1 expression and subcellular localization. Immunofluorescence staining for SUMO1 (green) and synaptophysin (red) for (**A**) Non-Tg and (**B**) SUMO1 Tgs in the dentate gyrus and (**C**) Non-Tg and (**D**) SUMO1-Tg mice in the CA3 layer. Localization of transgene expression investigated by double-labeling in the CA3 layer (**E**) for neuronal marker NeuN (green) and SUMO1 (red) in Tg animals. (**F**) Staining for astrocytic GFAP (red) indicated a co-localization with SUMO1 (green) within glia (arrows). SUMO1 staining in A-D was performed with the rabbit SUMO1 polyclonal and commercial monoclonal antibody (Cell Signaling) in E-F. Scale bars 50 μm.

Subcellular localization was assessed by immunofluorescence using the rabbit polyclonal antibody which showed nuclear staining for SUMO1 in both the Tgs and non-Tg mice (Fig. 2A,B). This is consistent with its predominant nuclear localization and function. However, staining was not limited to the nucleus as SUMO1 was also found in neuronal processes and cytoplasmic compartments which was seen in non-Tgs and enhanced in the Tg animals (Fig. 2B,D). This was seen in all brain regions as indicated by staining in the dentate gyrus and CA3 layer (Fig. 2A-D). It was difficult to quantify the level of expression by immunofluorescence as the polyclonal antibody displayed high affinity and therefore comparable intensities were seen in both SUMO1 Tgs and non-Tg mice. However, utilization of commercially-available SUMO1 antibodies clearly revealed a distinction as staining was only observed in the SUMO1-Tg animals and only a weak signal was detected in the non-Tg mice (Fig. S3).

Co-localization with synaptophysin was not as readily evident as compared to the nuclear staining which raises the question of whether or not the transgenic SUMO1 is trafficked to synapses (Fig. 2A-D). This is likely due to the high-intensity staining for SUMO1 in the nucleus but western blotting of isolated synaptosomes clearly demonstrated the presence of SUMO1 in both unconjugated and conjugated forms (Fig. S2). Double-labeling with the neuronal NeuN marker confirmed overlap with SUMO1 in the Tg mice (Fig. 2E). Similar co-staining with GFAP indicated virtually no overlap with SUMO1 as expected for the neuronal expression via the prion cos-tet vector (Fig. 2F). Cumulatively, these findings confirm the establishment of a SUMO1 Tg mouse with high levels of expression in neurons throughout the brain.

### **S**UMO1 Neuronal Conjugation Targets

**Figure 3:**
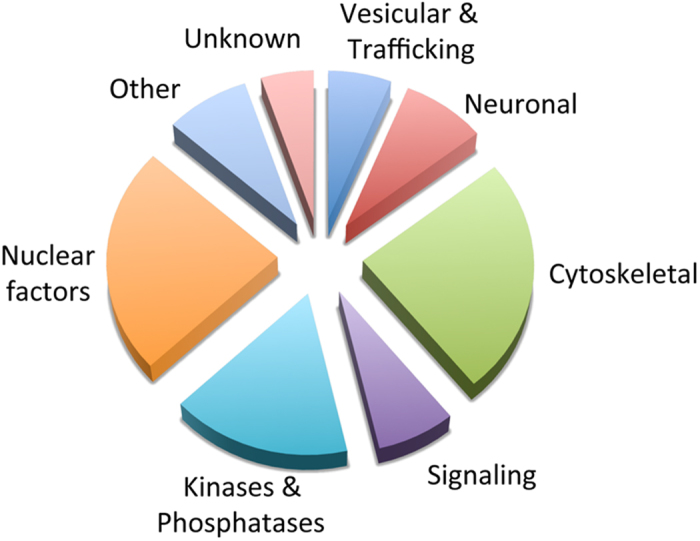
Identification of SUMO1 conjugation candidates. Proteomic analysis of putative SUMO1 conjugates. Large-scale immunoprecipitation and mass spectrometry analysis revealed approximately ~95 SUMO1 conjugation candidates. Classification and relative abundance of SUMO targets in different categories are indicated.

SUMO1 expression in the Tgs resulted not only in increased monomeric protein but a proportional elevation in the high molecular weight conjugates. Bulk immunoprecipitation with a SUMO1 polyclonal antibody was used to identify the conjugation targets from whole brain extracts of the SUMO1 Tg mice at 4 months of age. A comparable immunoprecipitation using I gG from a non-immunized rabbit was conducted on the same lysate as a control. The immunoprecipitates were run on SDS-PAGE, and proteins identified with Coomassie staining and in-gel trypsin digestion. The resulting peptides were identified using tandem mass spectrometry, and revealed ~95 unique SUMO1 conjugation targets with high confidence from a variety of different protein classes (Dataset S1). Expected nuclear and transcription factors were identified as well as a variety of kinases, phosphatases and ATPases (Fig. 3). In addition, a significant number of vesicle-related and trafficking proteins were observed, many of which were neuron- and synapse-specific such as synapsin-1 and -2 as well as synaptic vesicle glycoprotein 2A and synaptosomal-associated protein 25.

**Figure 4:**
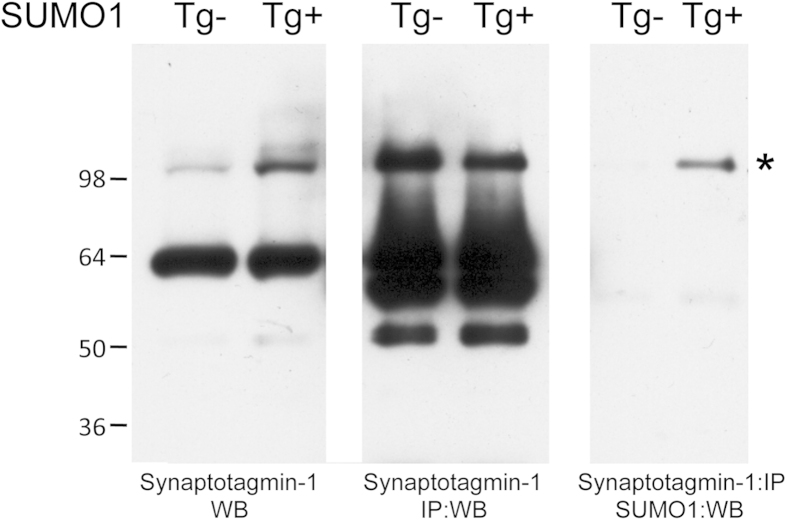
Validating neuronal SUMO1 conjugates. Whole brain lysates probed for synaptotagmin-1 in Non-Tg (Tg-) and SUMO1 transgenes (Tg+) with immunoreactivity present for the full-length protein (~64 kD) and high molecular weight species (~100 kD) (left panel). Immunoprecipitation of synaptotagmin-I followed by western blotting of the protein revealed strong reactivity for both bands (middle panel). Identification of SUMO1 conjugated synaptotagmin-I was validated by the immunoprecipitation of synaptotagmin-I followed by western blotting for SUMO1 (right panel). Based on molecular weight changes synaptotagmin-I is predicted to contain either two SUMO on distinct lysine residues or a growing of a polymeric SUMO chain on a single lysine amino acid.

Interestingly, synaptotagmin-1 was uniquely identified in our Tgs. The SUMOylation of synaptotagmin-1 was examined directly by immunoprecipitation and western blotting to validate the proteomic findings. Total brain homogenates from SUMO1 Tgs and non-Tg mice were initially probed for synaptotagmin-1 which indicated the expected band at ~65 kD in both cases (Fig. 4). An additional higher molecular weight band was observed at ~100 kD which was most prominent in the SUMO1 Tgs but also detectable in the non-Tg animals. Immunoprecipitation of synaptotagmin-1 and re-probing gave the expected high intensity reactivity for the native protein but also strong reactivity for the higher molecular weight species (Fig. 4). A comparable immunoprecipitation of synaptotagmin-1 followed by western blotting with SUMO1 confirmed the modification of synaptotagmin-1 which was also observed in the non-Tg brain extracts with longer exposure (data not shown). These findings suggest that synaptotagmin-1 is natively SUMOylated, which is up-regulated in the Tg mouse model, possibly leading to an alteration in protein function.

### SUMO1 Overexpression Impairs Synaptic Function

**Figure 5:**
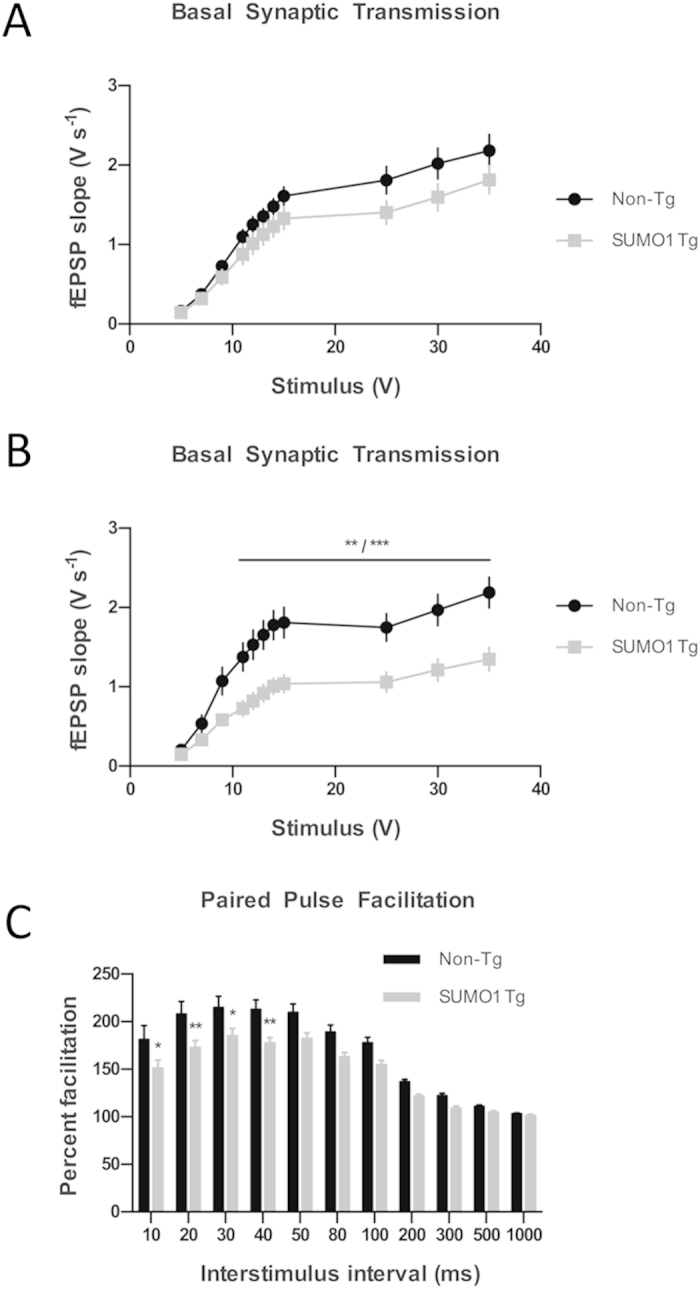
SUMO1 overexpression impairs synaptic function. (**A**) Input-output relationship plots of hippocampal slices from SUMO1 Tg and Non-Tg (4–8 months old). SUMO1 n = 46 slices (18 mice), Non-Tg n = 48 slices (20 mice); ANOVA p = 0.11. (**B**) Input-output relationship plots of hippocampal slices from SUMO1 Tg and Non-Tg mice (9–10 months old). SUMO1 n = 15 slices (8 mice), Non-Tg n = 12 slices (6 mice); ANOVA p = 0.003, multiple comparisons **p < 0.01, *** p < 0.001. **(C)** Paired pulse facilitation in hippocampal slices from SUMO1 Tg and Non-Tg mice. SUMO1 n = 34 slices (14 mice), Non-Tg n = 28 slices (12 mice); ANOVA p = 0.003, *p < 0.05, **p < 0.01. Data are presented as mean ± SEM.

To investigate the potential effects of SUMO1 overexpression on synaptic functioning, we performed field potential recordings with acute hippocampal slices from SUMO1 Tg mice. In younger adult mice (4–8 months old), we observed a trend towards decreased basal synaptic transmission, as assessed by input-output relationship plots (Fig. 5A). However, the trend did not reach statistical significance in this age group. In older SUMO1-overexpressing mice (9–10 months old), the basal transmission deficit was statistically significant at stimulus intensities of 11–35 V compared to non-Tg littermate controls (Fig. 5B). These findings indicated a progressive decline in total input-output measures which could reflect a decrease in synaptic activity and/or a loss of synapses.

Since synaptotagmin-1 is particularly important for presynaptic function, we performed paired pulse facilitation (PPF) measurements. PPF is a form of short-term synaptic plasticity likely dependent upon presynaptic mechanisms – in general, PPF involves the facilitation of neurotransmitter release caused by residual calcium from a previous stimulus. We observed that SUMO1 Tg mice (4–8 months old) exhibited significantly impaired PPF (Fig. 5C); at the low interstimulus intervals of 10–40 ms, SUMO1 Tg mice had significantly less facilitation compared to non-Tg littermate controls.

We conclude that SUMO1 overexpression results in an age-dependent reduction of basal synaptic transmission along with an earlier impairment of presynaptic function as suggested by the PPF results. These findings indicate that SUMO1 overexpression affects synaptic transmission through mechanisms responsible for the age-dependent reduction in basal synaptic function and the earlier impairment in PPF.

### Dendritic Spine Density is Reduced by SUMO1 Over-expression

The input-output data may not only be the result of altered function but may also reflect changes in neuronal morphology^9^. The proteomic analysis of the putative SUMO1 conjugates indicated a number of cytoskeletal proteins such as microtubule associated tau protein and neurofilaments (Table 1 and Fig. 3). Several high confidence proteomic candidates are also linked to synaptic stability and actin remodeling including spectrin α-chain and dihydropyrimidinase-related protein 2 (DRP-2) which are involved in axonal outgrowth and growth cone collapse (17). Interestingly a number of actin-related protein 2/3 (Arp2/3) complex components were identified such as FAM21, G protein-coupled receptor kinase-interactor 1 (Git1), cytoplasmic FMR1-interacting protein 1 (Cyfip1) and Rho guanine nucleotide exchange factor 7 which are key factors in dendritic spine formation^17^,^18^,^19^,^20^. The potential effects of SUMO conjugation on spine density and morphology were therefore assessed using Golgi staining approaches.

**Figure 6:**
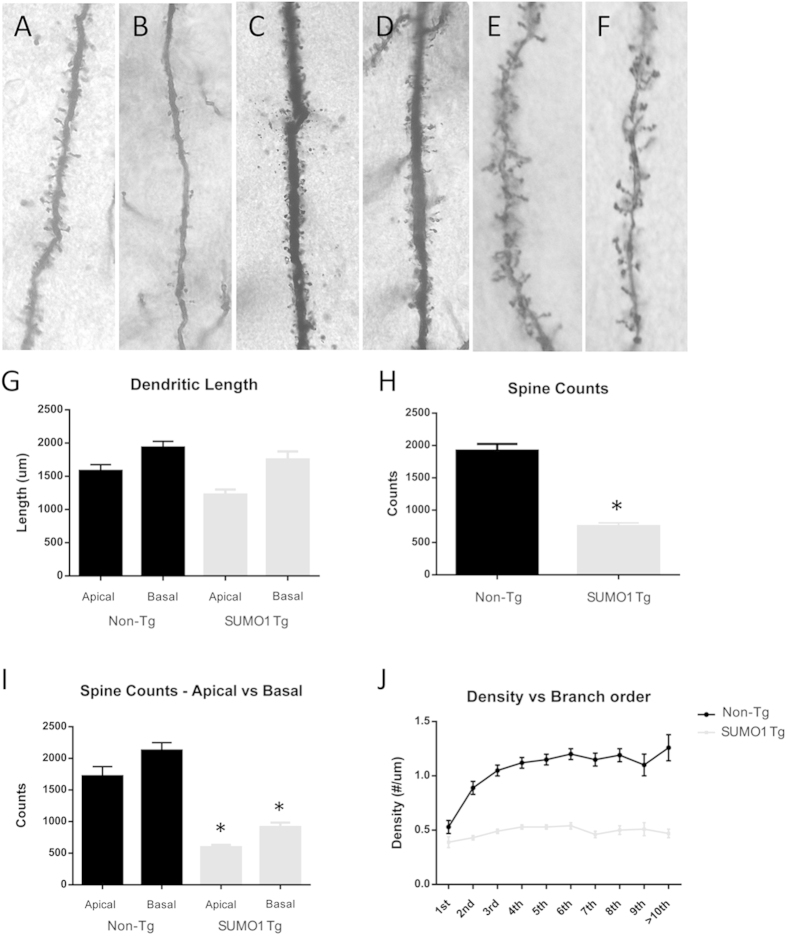
SUMO1 overexpression reduces dendritic spine density. Golgi staining was used to visualize neuronal processes which at lower magnification indicated normal spine formation and density in the frontal cortex pyramidal neurons of (**A**) Non-Tg mice which were significantly decreased in (**B**) SUMO1 Tgs. Analysis of the hippocampus CA1 pyramidal neurons of (**C**) Non-Tg and (**D**) SUMO1-Tg mice indicated a similar trend. Higher magnification of dendritic spines in the (**E**) Non-Tg mice compared to (**F**) SUMO1 Tg animals reveals similar morphology but reduced density (all analyses performed with n = 3; SUMO1 Tg and Non-Tg). (**G**) Measurements of spine lengths in apical and basal dendrites of frontal cortex pyramidal neurons indicated non-significant differences in Non-Tg versus SUMO-Tg mice (p > 0.05). (**H**) Total spine counts of pyramidal cells confirmed a ~70% decrease in the SUMO1 Tgs (SUMO1 n = 22,617 spines, Non-Tg n = 57,608 spines; *p < 0.05). (**I**) Spine number was equally reduced in the SUMO1 mice in apical (SUMO1 n = 8,927 spines, Non-Tg n = 25,762) and basal (SUMO1 n = 13,690 spines, Non-Tg n = 31,846) dendrites of pyramidal cells spines (*p < 0.05). (**J**) Analysis of spine counts for pyramidal neurons in the frontal cortex as a function of branch order indicated a uniform reduction in the SUMO1 mice along all dendritic zones (*p < 0.05).

Examination of dendritic spine numbers of neurons indicated a qualitatively significant decrease in density in the SUMO1 Tgs as compared to non-Tg animals (Fig. 6). Pyramidal neurons in the frontal cortex had dramatically reduced spine densities in the Tgs when visualized at low magnification (Fig. 6A,B). Similar reductions were observed in CA1 hippocampal neurons suggesting a broadly distributed effect in the SUMO1 over-expressing mice (Fig. 6C,D). At higher magnification, the spine lengths and morphology were comparable in the two sets of animals suggesting assembly was not compromised but mainly spine numbers (Fig. 6E,F).

Quantification of spine density in the cortical pyramidal neurons revealed an ~70% reduction in the SUMO1 transgenic animals (Fig. 6H). The decrease was not restricted to a particular dendritic zone as indicated by similar changes in both apical and basal dendritic spines in pyramidal cells (Fig. 6I). More detailed quantification along the branching order demonstrated the typical increase in spine number with high branch orders which was not seen in the SUMO1 Tg mice (Fig. 6J). Finally, the decrease in spine density of SUMO1 overexpressing mice was not accompanied by significant changes in spine length in both apical and basal dendrites (Fig. 6G).

### SUMO1 Transgenics Display Memory Impairment

**Figure 7:**
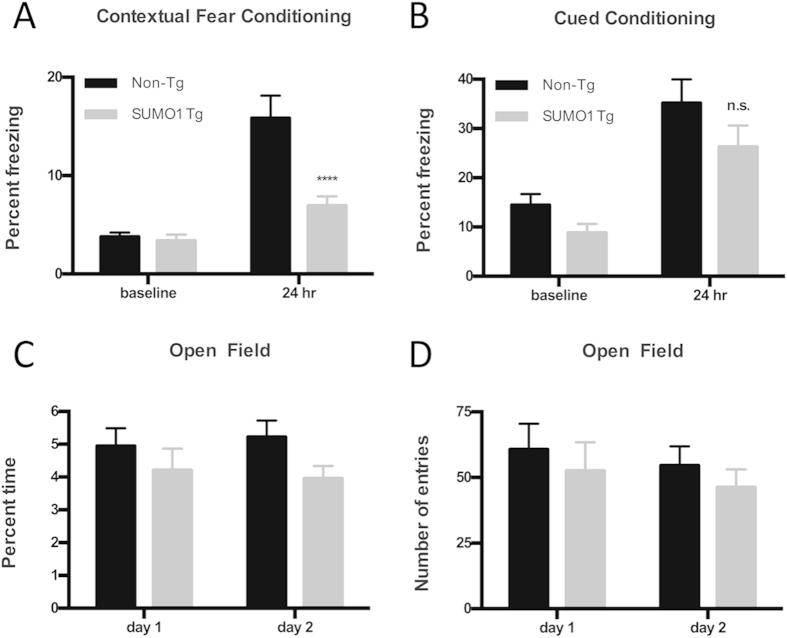
Contextual Fear Memory is Impaired in SUMO1 transgenic mice. (**A**) Contextual fear conditioning performed 24 hours after training shows a reduction in freezing for the SUMO1 Tgs compared to Non-Tgs. SUMO1 n = 23 animals (all males), Non-Tg n = 21 animals (all males) in this and the following panels. ****p < 0.0001. (**B**) No significant differences were observed for cued conditioning between Non-Tg and SUMO1 Tg mice. Open field testing revealed similar behaviors with respect to (**C**) number of entries and (**D**) percent time between the two groups of mice.

The observed changes in synaptic transmission and dendritic spine loss might have had a broader impact on learning and memory in the SUMO1 Tg mice. Contextual and cued fear conditioning (FC) was therefore investigated to determine if the SUMO1 over-expressing mice had a distinguishable behavioral phenotype. Testing of contextual FC, a type of associative memory depending upon hippocampal and amygdala function^21^ in 6–8 month old animals revealed an impairment in memory where SUMO1 Tg mice displayed a significantly reduced freezing time 24 hrs after conditioning (Fig. 7A). However, cued FC testing, a behavioral task assessing amygdala function, indicated that there were no statistically significant differences between Tg and non-Tg mice over the same conditioning period (Fig. 7B). These observed memory impairments were not due to physiological difference in the perception of the electric shock resulting from SUMO1 overexpression as both groups, for example, displayed identical sensory thresholds (Fig. S4). The specific effect seen in contextual FC would be therefore consistent with a specific hippocampal involvement and may reflect a greater impact of SUMO1-mediated changes in this brain region.

Interestingly, the SUMO1 Tg mice did not display other behavioral phenotypes and no changes in anxiety as demonstrated by similar open field responses. This was the case for both number of entries and percent time in the open (Fig. 7C,D). The SUMO1 Tgs also had no readily apparent changes in motor function and appeared to be grossly normal when compared to non-Tg animals.

## Discussion

There is mounting evidence from a number of recent studies that point to a role of SUMO modification in neuronal function and neurodegenerative diseases^8^,^22^. However, only a limited number of targets have been identified, primarily through *in vitro* approaches, and there are potentially many more SUMO conjugated candidates to be identified. Moreover, the role of SUMO in neuronal function and behavior needs to be fully characterized. The current study was focused onto SUMO1, one of three primary SUMO paralogs. Specifically, we characterized an *in vivo* SUMO1 Tg mouse model to investigate neuronal targets and their effects on synaptic function, neuronal morphology, and memory. Proteomic analysis has led to the identification of a number of previously unrecognized SUMO1 candidate proteins linked to a variety of neuronal pathways. High levels of SUMOylation resulted in impaired synaptic transmission, changes in neuronal morphology, and memory loss.

Previous investigations have identified several synaptic proteins that undergo SUMOylation and may impact neuronal function and disease-related pathways. Among the ones characterized in neurons and/or brain tissue are the myocyte enhancer factor 2A (MEF2A) transcription factor^23^, the kainate receptor subunit GluR6^24^, the RNA-binding protein La^25^, the cannabinoid receptor type 1 (CB1)^26^, the immediate early gene Arc^27^ and the calcium/calmodulin-dependent serine protein kinase (CASK)^28^. SUMOylation of these proteins was found to regulate a variety of neuron-specific functions, including postsynaptic differentiation (MEF2A), receptor endocytosis (GluR6), axonal transport (La), homeostatic synaptic scaling (Arc), and spine morphogenesis (CASK). Our investigation of the SUMO1 Tg mice has identified a number of additional protein targets related to synaptic function and morphology (Dataset S1 and Table 2), including nuclear and transcription factors, a variety of kinases, phosphatases and ATPases, as well as several vesicle-related and trafficking proteins.

At the synapse, it is likely that both the pre- and post-synaptic compartments contain SUMOylated proteins. The SUMO enzymatic machinery is also present throughout neurons and can undergo activity-dependent redistribution^12^,^24^. Pre-synaptic SUMOylation has been shown to modulate glutamate release and to regulate calcium influx, an essential process involved in vesicle fusion at the presynaptic membrane^29^,^30^. In our studies, SUMOylation of one of the more significant presynaptic candidates, synaptotagmin-1, was found to be upregulated. Synaptotagmin-1 has been associated with regulation of neurotransmitter release^31^. Its overexpression positively modulates short-term synaptic plasticity at developing neuromuscular junctions^32^. This would be consistent with the observed impairment in PPF in the SUMO1 Tg mice. An attractive hypothesis is that increase in SUMOylation of synaptotagmin-1 by SUMO1 impairs the protein positive modulation of PPF.

A significant reduction in dendritic spine density was observed in the SUMO1 Tg mouse. This may be due to modification of MEF2A for which precedents have been reported for its role in neuronal arborization and synaptic development^23^,^33^. In this case, proteomic analysis of the immunoprecipitated SUMO1 target proteins from total brain did not identify MEF2A as a target protein. This does not suggest that it is not modified as it may be rapidly turned over or present in lower abundance. However, a number of SUMO1-conjugated targets linked to dendritic spine formation were identified in the SUMO1 transgenic mice. For example, the G protein-coupled receptor kinase-interactor 1 (GIT1) which is a member of the Arp2/3 complex responsible for actin remodeling within synapses^34^,^35^. Genetic knockout of GIT1 in mice results in a dramatic reduction in spine density and impaired fear response similar to that observed in the SUMO1 animals^36^,^37^. SUMOylation of GIT1 may therefore represent one potential mechanistic pathway leading to the observed changes in dendritic spines in the SUMO1 mice.

A recent SUMO1 knock-in mouse model has been described that incorporated homozygous His6-HA-SUMO1 to maintain endogenous levels of expression while retaining the ability to isolated conjugated proteins^38^. SUMO1 was localized primarily to nuclear compartments which was consistent with the novel targets identified in these knock-in mice by proteomic analyses. However, virtually no synaptic localization was observed for the His6-HA-SUMO1 which could possibly be due to lower abundance, lack of stimulus to promote trafficking and/or a rapid turnover of SUMO1 conjugates outside of the nucleus. The knock-in approach represents a more physiological model of SUMOylation and avoids identification of potential off-target protein modifications that may result from abnormally high levels SUMO1 expression. This is a valid concern with SUMO1 over-expressing transgenic models described in the current study and further investigation of the target identified is going to be required. Nevertheless, this concern is somewhat alleviated by the observation that elevated SUMO1 expression in our Tg mice had relatively little effect on the other SUMO proteins or the conjugation pathway elements. It is therefore conceivable that the current model primarily reflects an alteration in function for many of the targets identified as opposed to artifacts resulting from elevated SUMO1 in neurons. The complementary approaches of the knock-in and Tg models may therefore provide a greater understanding as to the pathways impacted by SUMO1 and their consequences.

The findings from the Tg approach demonstrate that SUMO1 has profound effects on synaptic function that culminate in impaired cognition. Alterations in neuronal SUMO1 modification may therefore have wider implications on mental retardation and related neurological disorders. In addition, SUMOylation has been linked to a number of neurodegenerative diseases such as Alzheimer’s and Parkinson’s disease. The current transgenic model may provide further opportunities to elucidate how SUMO1 may contribute to the pathologies of these diseases.

## Methods

### Construction of the SUMO1 transgenic mouse model

All protocols involving animals were approved by the University of Toronto and Columbia University and the respective Institutional Animal Care and Use Committee (IACUC); experiments involving animals were performed in accordance with the relevant approved guidelines and regulations. Human SUMO1 cDNA was isolated from a plasmid (pcDNA3) encoding the complete sequence of SUMO1, as previously described^6^. Digestion with Sma I and Spe 1 produced two fragments of 90 and 269 base-pairs, which included the 5’ and 3’ untranslated (UTR) regions. These fragments were cloned into the plasmid vector pUC19 and subjected to mutagenesis using the “transformer” protocol (Clontech), in order to convert the 5’ Sma I site to a Sal I site, with a second Sal I site deriving from the pUC19 polylinker. A ~2.4 Kb fragment was excised by digestion with Sal I and inserted into the Sal I cloning site of pBR322, to exclude extraneous polylinker sites and thereby facilitate swapping of internal SUMO1 restriction fragments (“Quick-change”, Stratagene Inc.). The resulting plasmid was sequenced in its entirety with a total of 12 sequencing primers to cover the SUMO1 coding region and exclude the presence of mutations. Not I transgene fragments excised from this cosmid vector were purified and transgenics generated by standard pronuclear injections at the McLaughlin Research Institute (Great Falls, MT). Founder animals were identified by dot-blot hybridization analysis of genomic DNA using a probe within the hamster PrP gene 3’ UTR as described previously^39^. The SUMO1 transgenics were maintained on a mixed C57Bl6/C3H/FVB background.

### Generation of SUMO antibodies

Peptide antigens used for polyclonal antibody generation corresponded to SUMO1 C-terminus residues 73–97 (IADNHTPKELGMEEEDVIEVYQEQT) and SUMO2/3 N-terminal residues 3–24 (EEKPKEGVKTENDHINLKVAGQ). Peptides were covalently linked to keyhole limpet hemocyanin (KLH) and used to immunize rabbits. Polyclonal antibodies from collected and pooled antisera were affinity-purified as previously described^40^.

### Immunofluorescence staining

Half brains of 22 week old male and female Non-Tg and SUMO1-Tg mice were dissected into 10% buffered formalin and kept at 4 ^o^C overnight. Samples were then embedded in paraffin and sagittal sections were probed with primary antibodies corresponding to rabbit anti-SUMO1 (1:1000), mouse anti-SUMO1 (1:100; MAB 5718 Cell Signaling Technology), mouse anti-Synaptophysin (1:200; 61880 BD Biosciences), rabbit anti-NeuN (1:200; ABN78 Millipore), and mouse anti-GFAP (1:100; MAB 3670 Cell Signaling Technology). Alexafluor 488-conjugated anti-rabbit (1:1000; A20216 Invitrogen) and Cy3-conjugated anti-mouse (1:1000; 115–165-146 Jackson ImmunoResearch) were used as secondary antibodies.

### Immunoprecipitation

Whole brain samples from Non-Tg or SUMO1+/+ Tg mouse were homogenized in lysis buffer (50 mM HEPES-KOH pH 8.0, 100 mM KCl, 2mM EDTA, 0.5% NP-40, 10% glycerol and protease inhibitors). Lysates were next incubated for 30 minutes at 4 °C, samples were centrifuged at high speed (15,000 g) and supernatants were collected. Protein lysates were first subjected to pre clearing with protein G-Sepharose 4 fast flow (GE Healthcare). SUMO1 polyclonal antibody (400 μg total) or preimmune rabbit serum conjugated beads were added to 120 mg of non-Tg or SUMO Tg mouse total brain lysates. Immunoprecipitated proteins were elutated with in Laemmli buffer and resolved by SDS-PAGE (4–12% Bis-Tris gels, BioRad) before proteomic analyses. Gels were stained with Coomassie brilliant blue for visualization and the entire protein-containing gel piece was processed for mass spectrometry as previously described^41^.

### Mass spectrometry

Samples were re-suspended in 0.1% MS grade formic acid (Sigma). Analytical columns (75 μm inner diameter) and pre-columns (100 μm) were prepared in-house from silica capillary tubing (InnovaQuartz, Phoenix, AZ), and packed with 5 μm 300 Å C18-coated silica particles (Michrom). Peptides were subjected to nLC-ESI-MS/MS, using a 120 min reversed phase (10–40% acetonitrile, 0.1% formic acid) buffer gradient running at 250 nL/min on a Proxeon EASY-nLC pump in-line with a hybrid linear quadrupole ion trap (Velos LTQ) Orbitrap mass spectrometer (Thermo Fisher Scientific). A parent ion scan was performed in the Orbitrap, using a resolving power of 60,000. Simultaneously, up to the forty most intense peaks were selected for MS/MS (minimum ion count of 1000 for activation) using standard CID fragmentation. Fragment ions were detected in the LTQ and dynamic exclusion was activated such that MS/MS of the same *m*/*z* (within a 10 ppm window, exclusion list size 500) detected two times within 15 sec were excluded from analysis for 30 sec. Thermo .RAW files were converted to the .mzXML file format^42^ and searched with X!Tandem^43^, using a parent mass window of 15 ppm and 0.4 Da fragment mass window, against the combined human and mouse Fasta databases (Ensembl). Up to two missed cleavages were allowed. Oxidation of methionine, and deamidation of glutamine and asparagine were set as variable modifications. Proteins identified with two or more spectral counts, and with log(e) expect score < −2 (and which were not identified in the control experiment) are shown in Supplemental Dataset S1.

### Morphological studies

For spine density and morphological analyses, FD Rapid GolgiStain Kit (FD NeuroTechnologies) was used for both SUMO1 Tg and Non-Tg animals. Sections were prepared using a vibratome with a thickness of 150 μm. Dissected mouse brains were submerged in 5% potassium dichromate (Solution A) and 5% mercuric chloride (Solution B) for 2 weeks at room temperature and transferred to 5% potassium chromate (Solution C) for 24 h at 4 °C. Dendritic spine density counts and morphological assessments were performed under bright-field microscopy.

### Electrophysiological studies

Transverse hippocampal slices (400 μm) were cut with a tissue chopper and maintained in an interface chamber at 29 °C for at least 90 minutes prior to recording, as previously described^44^. The artificial cerebrospinal fluid (ACSF) bath buffer consisted of (in mM): 124 NaCl, 4.4 KCl, 1 Na_2_HPO_4_, 25 NaHCO_3_, 2 CaCl_2_, 2 MgCl_2_, 10 glucose. The buffer was continuously aerated with 95% O_2_ / 5% CO_2_ to a final pH of 7.4. Field extracellular postsynaptic potentials (fEPSPs) were recorded using a stimulating electrode (bipolar tungsten) at the CA3-CA1 Schaffer collateral fibers and a recording electrode (ACSF-filled glass pipette) at the CA1 *stratum radiatum*. Basal synaptic transmission was assessed by plotting stimulus voltages (V) against the corresponding fEPSP slopes. Stimulus intensity was set so that baseline responses were approximately 1/3 of the maximum evoked response. Paired pulse facilitation (PPF) was elicited through two successive stimuli, separated by an inter-stimulus interval, and measured as a ratio of the two fEPSP slopes. All experiments were performed with interleaved controls.

### Behavioral studies

#### Fear conditioning (FC)

FC was performed as previously described^45^. Briefly, mice were placed in a conditioning chamber for 2 minutes. Subsequently, a 30 second tone sound was played and a 2 second foot shock (0.8 mA) was administered at the end of the tone. The mice were left in the chamber for an additional 30 seconds. After 24 hours, the mice were placed back in the same chamber, and freezing behavior was scored using EthoVision XT software; freezing behavior was defined as the absence of movement except for breathing. Contextual fear memory was assessed over 5 minutes in the chamber. Cued fear learning was assessed 24 hours after the contextual assessment by placing the mice in a novel context for 2 minutes (pre-tone) and then playing the training tone sound for 3 minutes (post-tone). Sensory thresholds were assessed by measuring the minimal foot shock intensities at which a mouse manifested a behavioral response in three categories: visible response to shock (flinching), extreme motor response (jumping) and vocalized distress (screaming).

#### Open Field

Open field testing was performed as previously described^45^. Briefly, mice were placed in an open arena situated in a dim room. The dimensions of the arena were 27.3 (L) x 27.3 (W) x 20.3 (H) cm. A digital camera was located above the arena and connected to the EthoVision XT tracking system. Scoring evaluations were based on the number of entries into and the percentage of time spent in the central compartment, over 10 minute sessions for two consecutive days.

#### Statistical analyses

All data are presented as means ± SEM. Data were analyzed by ANOVA plus post-hoc Sidak multiple comparisons test using Prism (GraphPad) software. The threshold for significance was set at p < 0.05 in all analyses.

## Additional Information

**How to cite this article**: Matsuzaki, S. *et al.* SUMO1 Affects Synaptic Function, Spine Density and Memory. *Sci. Rep.* **5**, 10730; doi: 10.1038/srep10730 (2015).

## Supplementary Material

Supplementary Information

Supplementary Information

## Figures and Tables

**Table 1:** Cytoskeletal and dendritic spine SUMO1 target proteins.

**SUMO1 Target**	**Log(e)**	**Function**	**References**
Cytoplasmic FMR1-interacting protein 1 (Cyfip1)	−140.9	WAVE-Arp2/3 complex protein Actin remodeling, axonal outgrowth	19
D6Wsu116e, Protein FAM21	−88.2	Activates Arp2/3 complex Actin polymerization Wiskott-Aldrich Syndrome	46
Microtubule-associated protein tau	−36.1	Microtubule stability, axon polarity Alzheimer pathology	47
Dihydropyrimidinase- Related protein 2 (DRP-2)	−25.1	Neuronal development, axon growth, Neuronal growth cone collapse	48
Neurofilament heavy polypeptide (NF-H)	−24.1	Maintenance of neuronal caliber	49
Rho guanine nucleotide exchange factor 7(PAK-interacting exchange factor beta)	−9.8	CaMKK-CaMK1 signaling cascade Hippocampal spine formation	20

**Table 2:** Synaptic SUMO1-conjugation target proteins.

SUMO1 Target	Log(e)	Function	References
ARF GTPase-activatingprotein GIT1	–88.2	Vesicle trafficking, cytoskeletalorganization, spine formation	18,17
CaM-kinase II gamma LTP,hippocampal learning	–55.0	Dendritic spine/synapse formation	50
Synaptotagmin-1	–26.2	Presynaptic Ca2+ sensorneurotransmitter release	31
Synapsin-2 (Syn2)(Syn1)	–24.2–13.3	Synaptic vesicle componentsneurotransmitter release	51
