# Supplementary material for: SUMO1 Affects Synaptic Function, Spine Density and Memory
Source: Sci Rep. 2015 May 29;5:10730. doi: 10.1038/srep10730 (PMC4650663; doi:10.1038/srep10730)
Supplement: Supplementary Information [file srep10730-s1.pdf]

SUPPLEMENTARY INFORMATION

Title: SUMO1 Affects Synaptic Function, Spine Density and Memory

Authors: Shinsuke Matsuzaki[1,2,3,4], Linda Lee[5], Erin Knock[1], Tharan Srikumar[6,7], Mikako Sakurai[5], Lili-Naz Hazrati[1,7], Taiichi Katayama[2], Agnieszka Staniszewski[5], Brian Raught[6,7], Ottavio Arancio[5]* and Paul E. Fraser[1,7]*

Supplementary Figure S1. SUMO1 polyclonal antibodies were generated by immunization of rabbits with a KLH-conjugated peptide from the C-terminal domain (residues 73-97, sequence IADNHTPKELGMEEEDVIEVYQEQT). Ammonium-sulfate precipitated antibodies were affinity-purified using peptide antigen coupled to Sufo-link columns. **(A)** SUMO1 monomers and higher molecular weight conjugates were observed in HEK293 cells and immunoreactivity was confirmed with HA-tagged SUMO1 transfected cells. No bands were observed on western blots probed with preimmune serum (PI). **(B)** Antibody specificity was confirmed by western blotting of HEK293 cells transfected with HA-tagged SUMO2, 3 or 1 where only SUMO1 was recognized by the polyclonal antibodies.

Supplementary Figure S2. Examination of total brain extracts and isolated synaptosomes. **(A)** Immunoblotting demonstrated comparable levels of the SUMO activating enzyme E1 (SAE1) in the Non-transgenic (Tg-) and SUMO1 transgenic mice (Tg+). **(B)** Purified synaptosomes indicated higher levels of SUMO1 monomers and higher molecular weight conjugates in the transgenic animals similar to total brain lysates. Isolated synaptosomes for non-Tg and SUMO1 Tg mice demonstrated that SUMO1 was found at high levels in synapses that corresponded to monomeric and conjugated species.

Supplementary Figure S3. Immunoflourescence staining for NeuN (green) and SUMO1 (red) using the commercially available antibody in the CA3 area **(A,B)** and dentate gyrus **(C, D)** of Non-transgenic **(A,C)** and SUMO1 transgenic **(B,D)** animals. Scale bar = 50 μm.

Supplementary Figure S4. Sensory thresholds in non-transgenic (WT) and SUMO1 transgenic (SUMO1 Tg) mice were assessed by measuring the minimal foot shock intensities at which a mouse manifested a behavioral response in three categories: visible response to shock (flinching), extreme motor response (jumping) and vocalized distress (screaming). No significant differences were observed between the two groups of animals.

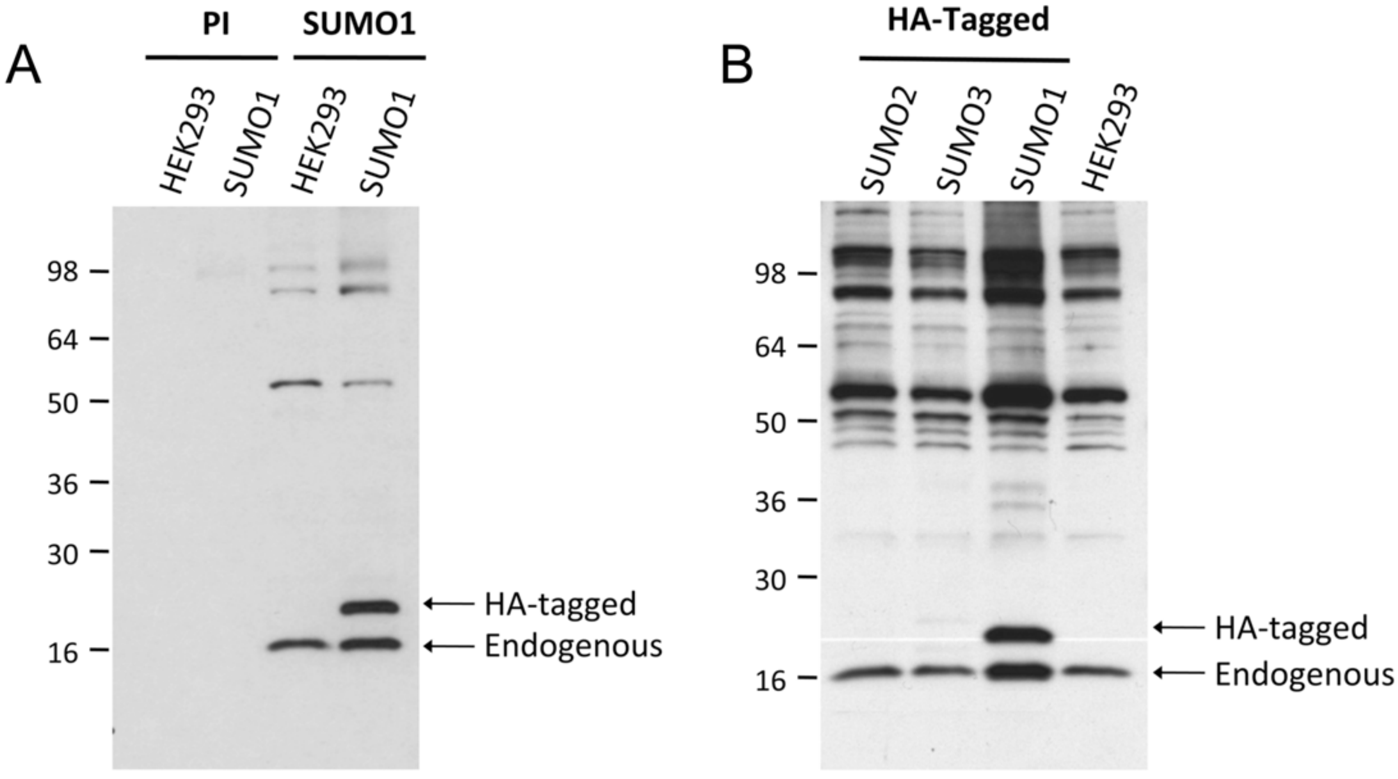
A
PI
SUMO1
HEK293
SUMO1
HEK293
SUMO1
98
64
50
36
30
16
HA-tagged
Endogenous
B
HA-Tagged
SUMO2
SUMO3
SUMO1
HEK293
98
64
50
36
30
16
HA-tagged
Endogenous

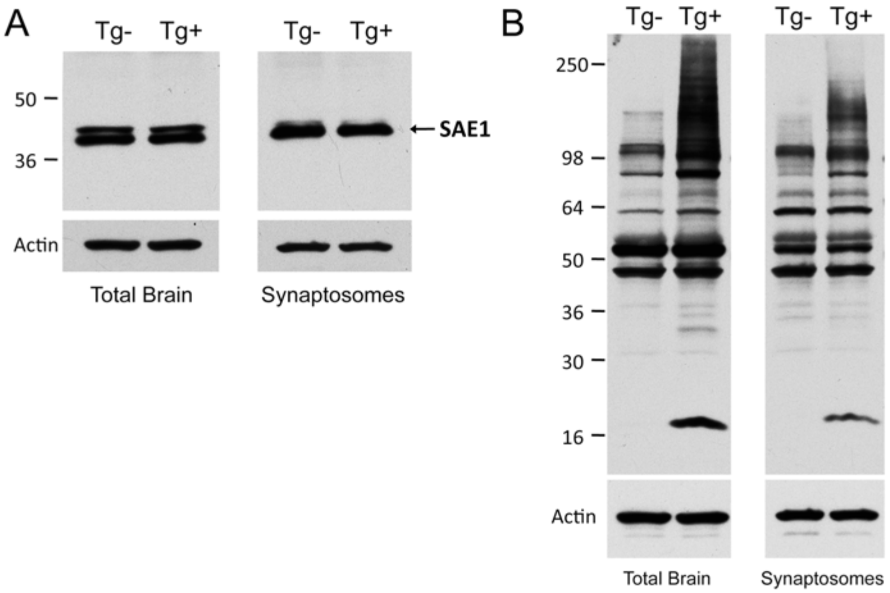
A
Tg-
Tg+
Tg-
Tg+
50
36
SAE1
Actin
Total Brain
Synaptosomes
B
Tg-
Tg+
Tg-
Tg+
250
98
64
50
36
30
16
Actin
Total Brain
Synaptosomes

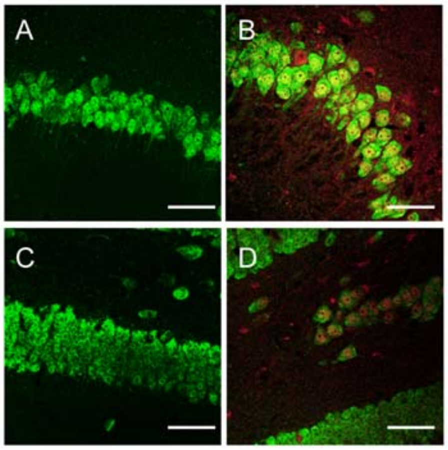
A
B
C
D

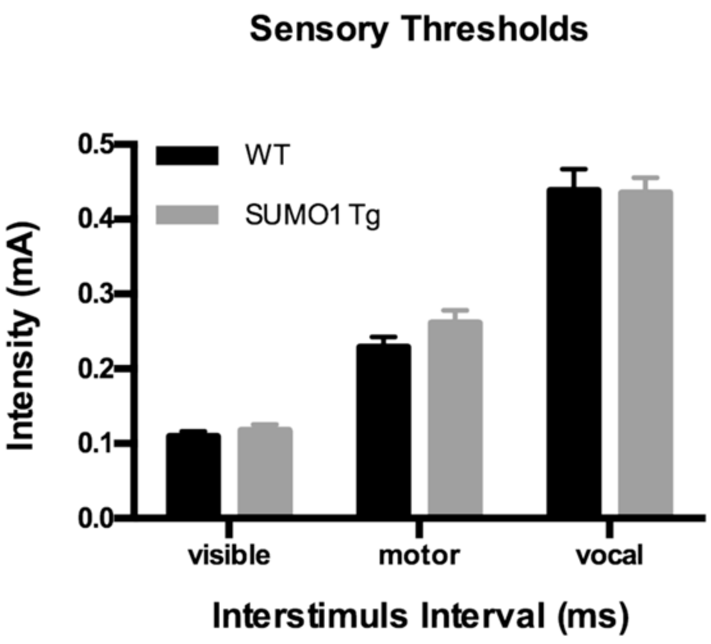
Sensory Thresholds
WT
SUMO1 Tg
Intensity (mA)
0.5
0.4
0.3
0.2
0.1
0.0
visible
motor
vocal
Interstimuls Interval (ms)
